# Benchmarking hybrid assemblies of *Giardia* and prediction of widespread intra-isolate structural variation

**DOI:** 10.1186/s13071-020-3968-8

**Published:** 2020-02-28

**Authors:** Stephen M. J. Pollo, Sarah J. Reiling, Janneke Wit, Matthew L. Workentine, Rebecca A. Guy, G. William Batoff, Janet Yee, Brent R. Dixon, James D. Wasmuth

**Affiliations:** 1 Department of Ecosystem and Public Health, Faculty of Veterinary Medicine, University of Calgary, Calgary, AB Canada; 2 Host-Parasite Interactions Training Program, University of Calgary, Calgary, AB Canada; 3 Bureau of Microbial Hazards, Food Directorate, Health Canada, Ottawa, ON Canada; 4 Department of Comparative Biology and Experimental Medicine, Faculty of Veterinary Medicine, University of Calgary, Calgary, AB Canada; 5 Division of Enteric Diseases, National Microbiology Laboratory, Public Health Agency of Canada, Guelph, ON Canada; 6 Department of Biology, Biochemistry and Molecular Biology Program, Trent University, Peterborough, ON Canada

**Keywords:** Long-read sequencing, MinION, *Giardia*, Structural variants, Heterozygosity, Parasite, Polyploidy, Tetraploid, Genome assembly

## Abstract

**Background:**

Currently available short read genome assemblies of the tetraploid protozoan parasite *Giardia intestinalis* are highly fragmented, highlighting the need for improved genome assemblies at a reasonable cost. Long nanopore reads are well suited to resolve repetitive genomic regions resulting in better quality assemblies of eukaryotic genomes. Subsequent addition of highly accurate short reads to long-read assemblies further improves assembly quality. Using this hybrid approach, we assembled genomes for three *Giardia* isolates, two with published assemblies and one novel, to evaluate the improvement in genome quality gained from long reads. We then used the long reads to predict structural variants to examine this previously unexplored source of genetic variation in *Giardia*.

**Methods:**

With MinION reads for each isolate, we assembled genomes using several assemblers specializing in long reads. Assembly metrics, gene finding, and whole genome alignments to the reference genomes enabled direct comparison to evaluate the performance of the nanopore reads. Further improvements from adding Illumina reads to the long-read assemblies were evaluated using gene finding. Structural variants were predicted from alignments of the long reads to the best hybrid genome for each isolate and enrichment of key genes was analyzed using random genome sampling and calculation of percentiles to find thresholds of significance.

**Results:**

Our hybrid assembly method generated reference quality genomes for each isolate. Consistent with previous findings based on SNPs, examination of heterozygosity using the structural variants found that *Giardia* BGS was considerably more heterozygous than the other isolates that are from Assemblage A. Further, each isolate was shown to contain structural variant regions enriched for variant-specific surface proteins, a key class of virulence factor in *Giardia*.

**Conclusions:**

The ability to generate reference quality genomes from a single MinION run and a multiplexed MiSeq run enables future large-scale comparative genomic studies within the genus *Giardia*. Further, prediction of structural variants from long reads allows for more in-depth analyses of major sources of genetic variation within and between *Giardia* isolates that could have effects on both pathogenicity and host range.
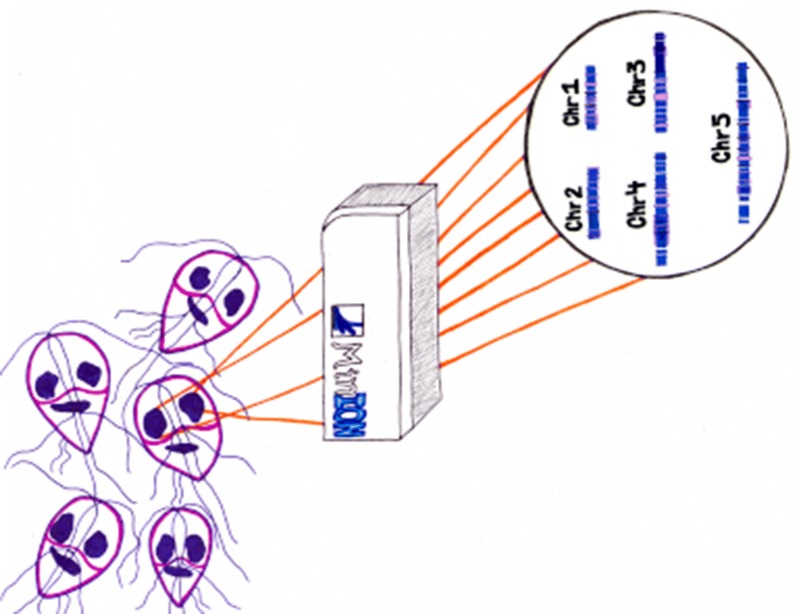

## Background

*Giardia intestinalis* (syns *Giardia lamblia* and *Giardia duodenalis*) is a single-celled, eukaryotic, food- and water-borne intestinal parasite that infects roughly 200 million people worldwide [1]. Infections can cause nausea, vomiting, diarrhea, and impaired growth and cognitive development [1]. The species *G. intestinalis* includes eight subtypes, named Assemblages A through H, at least two of which are known to infect humans (A and B) [1]. The cells have two diploid nuclei each containing five chromosome pairs [2]. The haploid genome size is ~ 12.8 Mb [3]. Genome comparisons amongst assemblages of *G. intestinalis* found only 77% nucleotide and 78% amino acid identity in coding regions, suggesting the assemblages may represent different species [4]. Six isolates of *G. intestinalis* have reference genomes available [3].

Currently, whole genomes are sequenced using second-generation technologies, third-generation technologies, or strategies involving combinations of technologies [5–7]. Second-generation sequencing platforms produce high quality reads with low error rates (0.1% for Illumina HiSeq) but short lengths (mean length < 250 bp for Illumina HiSeq), which pose challenges for assembly programs resulting in more fragmented assemblies [8]. In contrast, third-generation sequencing platforms produce much longer reads (mean length < 10,000 bp for PacBio and MinION) but have higher error rates (10–15% for PacBio and > 10% for MinION depending on the chemistry) [8–10]. These longer reads have the potential to resolve many genomic areas that are problematic for second-generation data, such as repetitive and/or duplicated regions [10]. Importantly, eukaryotic genomes have many such repetitive and duplicated regions (as much as two- thirds of the human genome may be repetitive elements [11]), making eukaryotic genomes especially good candidates for sequencing with third-generation technologies. Moreover, third-generation data are well suited for examining structural variants within a genome. In diploid and polyploid organisms, the different copies of each chromosome can contain large-scale differences relative to the consensus sequence that is generated during genome assembly, including insertions, deletions, duplications and translocations, in addition to variation at the single nucleotide level (SNPs). Polymorphisms greater than 100 bp are collectively called structural variants. They are a major source of genetic variation, thought to play a larger role in phenotypic variation than SNPs, but are difficult to resolve using second-generation data [12–14]. The tetraploidy of *Giardia* trophozoites further complicates short-read genome assembly and structural variant detection methods because of the increased computational complexity of constructing four haplotypes for each locus. For a review on the challenges associated with polyploid eukaryotic genomes see [15]. Our expectation is that long-read methods can detect and resolve the potentially three overlapping alternate alleles at any given locus.

The Oxford Nanopore Technologies (ONT) MinION is a third-generation sequencing platform based on nanopore technology [10, 16]. Briefly, the nucleic acids to be sequenced are driven through small pores in a membrane by an electrical current which causes fluctuations in the current in the pore [10]. Sensors measure these fluctuations, sending the data to a connected computer for processing and storage [10]. Assembling genomes *de novo* from MinION data involves basecalling of the squiggle files produced by the MinION during sequencing, assembly of the long reads into draft genomes, and polishing of the assemblies.

Here we have generated MinION and Illumina sequence data for *G. intestinalis* Assemblage A isolate WB (hereafter referred to as *Giardia* AWB), *G. intestinalis* Assemblage B isolate GS (hereafter referred to as *Giardia* BGS), and *G. intestinalis* isolated from a beaver (hereafter referred to as *Giardia* beaver). For each isolate, we assembled genomes from the long reads using several long-read assembler programs and evaluated each assembly on the basis of assembly metrics, gene finding, and comparison to the reference genome using whole genome alignments. We then added the short reads to the long-read assemblies to generate hybrid assemblies that were reference quality. After generating reference quality assemblies with the long and short reads, the long reads produced here were then used to investigate heterozygosity in each isolate by detecting the structural variants in each genome.

## Methods

### *Giardia intestinalis* isolates

*Giardia* AWB (ATCC 30957) and *Giardia* BGS (ATCC 50580) were obtained from the American Tissue Culture Collection, while *Giardia* beaver was a gift from Dr. Gaetan Faubert from McGill University, Canada. *Giardia* trophozoites were grown in TYI-S-33 medium [17] in 16-ml screw-capped glass tubes incubated at 37 °C.

### DNA extraction

Ten 16-ml culture tubes of each *Giardia* isolate (AWB, BGS and beaver) grown to late logarithm stage (~ 5–8 × 10^5^ cells/ml) were used for genomic DNA isolation. The culture tubes were chilled on ice for 5 min and the cells were collected by centrifugation at 1100×*g* for 15 min at 4 °C. Genomic DNA was extracted with DNAzol Reagent (Thermo Fisher Scientific, Waltham, USA) by following the manufacturer’s instructions. Briefly, each cell pellet was resuspended and lysed in DNAzol Reagent by gentle pipetting followed by a freeze (30 min at − 80 °C) and thaw (10 min at room temperature) step. The lysate was then centrifuged at 10,000×*g* for 10 min at 4 °C to remove insoluble cell debris. The supernatant was transferred to a new tube and the DNA was recovered by centrifugation of the supernatant at 4000×*g* for 5 min at 4 °C. The DNA pellet was washed twice with 75% ethanol then air-dried. The DNA was resuspended initially in 8 mM NaOH then neutralized by addition of HEPES to a final concentration of 9 mM.

RNA was removed from the DNA sample by the addition of 1–2 µl of 20 µg/µl RNase A (BioShop, Burlington, Canada) followed by incubation at 65 °C for 10 min. The degraded RNA was precipitated by the addition of ammonium acetate, incubation at 4 °C for 20 min and centrifugation at 12,000×*g* for 30 min at 4 °C. The supernatant was transferred to a new tube and the DNA was precipitated by the addition of 95% ethanol, incubation at room temperature for 5 min and centrifugation at 12,000×*g* for 20 min at 4 °C. The DNA pellet was washed once with 0.01 M ammonium acetate in 75% ethanol and once with 75% ethanol alone. The DNA pellet was air-dried before resuspension in TE buffer (10 mM Tris-HCl pH 8.0, 1 mM EDTA).

### MinION sequencing

The 1Dsq library preparation kit SQK-LSK308 was used as recommended by the manufacturer (Oxford Nanopore Technologies, Oxford, UK). Approximately 200 ng of prepared library was loaded onto a FLO-MIN107 (R9.5) flow cell. Data collection was carried out with live basecalling for 48 h, or until no more strands were being sequenced. All sequences were deposited in the sequence read archive (SRA) under accession number PRJNA561185.

### Illumina sequencing

Libraries were prepared using NexteraXT and paired-end sequenced on the MiSeq (v3, 2 × 300 cycles) or iSeq 100 (I1, 2 × 150 cycles) platforms according to manufacturer instructions (Illumina Inc., San Diego, USA). All sequences were deposited in the SRA under accession number PRJNA561185.

### Long-read basecalling, *de novo* assembly and genome polishing

Basecalling of all MinION output files was performed with the program Albacore (version 2.0.2) [18] using the full_1dsq_basecaller.py method to basecall both 1D and 1Dsq reads. The flowcell and kit parameters were FLO-MIN107 and SQK-LSK308, respectively.

*De novo* assemblies were performed using the programs Abruijn (version 2.1b) [19], Canu (version 1.6) [20] and SMARTdenovo (version 1.11 running under Perl version 5.22.0) [21]. Abruijn assemblies were conducted using the nanopore platform setting, coverage estimates calculated as the number of bases in the input reads divided by the reference genome size (Table 1) all rounded to the nearest integer, and all other default settings (one polishing iteration, automatic detection of kmer size, minimum required overlap between reads of 5000 bp, automatic detection of minimum required kmer coverage, automatic detection of maximum allowed kmer coverage). Canu assemblies were performed using Canu’s settings for uncorrected nanopore reads (-nanopore-raw), genome sizes estimated from the reference genome sizes (Table 1), and setting gnuplotTested=true to bypass html output report construction. SMARTdenovo assemblies were conducted using default settings (kmer length for overlapping of 16 and minimum required read length of 5000 bases).

**Table 1:** MinION sequencing run metadata, Albacore [18] basecalling results for both 1D and 1Dsq basecalling and read statistics

Name used in this document	AWB_0150	AWB_0157	AWB_2331	AWB_2338	Beaver_2302	Beaver_2309	BGS_2237	BGS_2244
Run name	SRRun1	SRRun1	SRRun2	SRRun2	SRRun3	SRRun3	SRRun4	SRRun4
Run ID	20170720_0150_GiardiaWB_20170719	20170720_0157_GiardiaWB_20170719	20170721_2331_GiardiaWB_20170721	20170721_2338_GiardiaWB_20170721	20170726_2302_GiardiaBeaver_20170726	20170726_2309_GiardiaBeaver_20170726	20170731_2237_GiardiaGS_20170731	20170731_2244_GiardiaGS_20170731
Isolate	*Giardia* AWB	*Giardia* AWB	*Giardia* AWB	*Giardia* AWB	*Giardia* beaver	*Giardia* beaver	*Giardia* BGS	*Giardia* BGS
Reference genome size (bp)	12,827,416	12,827,416	12,827,416	12,827,416	na	na	11,001,532	11,001,532
Sequencing depth (X genome size)	0.5	184.6	0.1	9.9	0.7	246.8	0.9	757.6
Total no. of 1D reads	1225	329,039	237	19,531	1668	382,740	1508	885,046
No. of 1D reads pass	1207	304,219	152	15,842	1603	354,581	1449	804,942
No. of 1D reads fail	18	24,820	85	3689	65	28,159	59	80,104
Percent of 1D reads passing	98.5	92.5	64.1	81.1	96.1	92.6	96.1	90.9
Total no. of IDsq reads	172	60,156	16	1904	146	53,553	212	143,371
No. of 1Dsq reads pass	68	25,755	0	192	69	29,349	124	62,452
No. of 1Dsq reads fail	104	34,401	16	1712	77	24,204	88	80,919
Average length of 1D reads	5066.15	7195.29	3450.08	6484.00	5113.00	8270.88	6534.03	9417.60
Longest 1D read	42,781	470,735	32,138	330,795	37,229	1,132,445	56,642	485,807
Average length of 1Dsq reads	5335.22	7685.61	2853.62	7344.74	5273.86	8472.84	5529.57	9829.82
Longest 1Dsq read	18,489	43,102	6523	32,705	22,740	59,564	25,876	66,185

*Notes*: “Pass” and “fail” refer to reads that met or did not meet the quality threshold, respectively. Run 2 was conducted on a previously used flow cell after 64–72 h run time and so had few pores left

*Abbreviations*: na, not applicable

Genome polishing is an error correction step performed on assemblies generated from third-generation data to compensate for the high error rate of the reads [10]. It involves re-evaluating the base calls from the MinION squiggle files together with the read overlap information from the assembly to improve base accuracy and correct small insertions and deletions [22]. Here, polishing was performed with the program Nanopolish (version 0.8.5) following the directions for “computing a new consensus sequence for a draft assembly” [23]. Briefly, the draft genome was first indexed using BWA (version 0.7.15-r1140) [24] and the basecalled reads were aligned to the draft genome using BWA. SAMtools (version 1.6 using htslib 1.6) [25] was then used to sort and index the alignment. Nanopolish then computed the new consensus sequence in 50 kb blocks in parallel, which were then merged into the polished assembly.

The commands used in the assembling and subsequent analyses can be found in Additional file 1: Text S1.

### Read error profile analysis

Read error profiles were examined for the six *Giardia* AWB and *Giardia* BGS runs using the program NanoOK (version v1.31) [26]. NanoOK extracts fasta sequences from the fast5 files produced by the MinION and aligns them to the reference genome using the LAST aligner (version 876) [27]. It then calculates error profiles for each set of reads that aligned to each contig in the reference. To obtain overall values for all reads in the sequencing run, for each error metric the value for each contig was extracted from the .tex file produced by NanoOK and multiplied by the proportion of the total reads mapping to that contig. These values were then summed to yield the metric value with respect to all reads in the sequencing run. The sums were scaled according to the proportion of the total reads that were included in the metric calculation (those that were mapped to the contigs) to yield the metric value for all reads used in the analysis.

### Long-read assembly evaluation

The effects on final assembly quality were evaluated for the following parameters: 1D *vs* 1Dsq input reads, pooling reads for the same organism from multiple runs, assembly program, and number of genome polishing iterations. First, 13 distinct input combinations, that represent all permutations of pooling runs for the same organism for both 1D and 1Dsq reads, were used for *de novo* assemblies: AWB_0157 1D reads; AWB_0157 1Dsq reads; AWB_0150_0157 1D reads; AWB_0150_0157 1Dsq reads; AWB_2338 1D reads; AWB_2338 1Dsq reads; AWB_2331_2338 1D reads; AWB_0150_0157_2331_2338 1D reads; AWB_0150_0157_2338 1Dsq reads; BGS_2244 1D reads; BGS_2244 1Dsq reads; BGS_2237_2244 1D reads; and BGS_2237_2244 1Dsq reads (Table 1). Each of these input combinations was used to perform a *de novo* assembly with each of the three assemblers used: Abruijn, Canu and SMARTdenovo. All of the resulting assemblies that produced contiguous sequences were polished with Nanopolish. Eight rounds of Nanopolish polishing were performed on the Canu and SMARTdenovo assemblies and seven rounds were performed on the Abruijn assemblies (which get polished once by Abruijn).

All assemblies and polished versions of the assemblies were aligned to the corresponding reference genome using the LAST aligner (version 876) [27] following the example for human-ape alignments [28]. Briefly, the reference genome was indexed using LAST, then substitution and gap frequencies were determined using the last-train method [29]. Finally, alignments were performed using the lastal method and the determined substitution and gap frequencies. The resulting alignments were then filtered to retain only those alignments with an error probability < 1e^−5^. *Giardia* AWB assemblies were aligned to only the contigs from the reference genome labelled GLCHR01, GLCHR02, GLCHR03, GLCHR04 and GLCHR05 (representing the five chromosomes of *G. intestinalis*). Filtered alignments were converted to other file formats (for metric calculation) using the maf-convert method in the LAST aligner.

Average percent identity was calculated from alignments in blasttab format by taking the sum of the percent identity multiplied by the alignment length for each aligned portion and dividing that sum by the total alignment length. Proportion of mismatching bases was calculated from alignments in psl format by taking the sum of mismatching bases for all aligned portions divided by the total alignment length. Total number of indels per 1000 aligned bases was calculated from alignments in psl format by taking the sum of the number of insertions in the query and the number of insertions in the target for all aligned portions, dividing that sum by the total alignment length and multiplying by 1000. Average size of indels was calculated from alignments in psl format by taking the sum of the number of bases inserted in the query and the number of bases inserted in the target for all aligned portions and dividing that sum by the total number of indels. The proportions of the reference covered 0, 1, 2, 3 or 4 times were calculated using BEDtools (version v2.27.1) [30]. Alignments were first converted to SAM format and SAMtools was used to sort the alignment and convert it to a bam file. The genomecov function of BEDtools was then used to analyze the coverage of every base in the reference genome in the alignment. The proportion of bases in the reference genome with 0, 1-, 2-, 3- and 4-fold coverage in the assembly were retrieved.

The assembly evaluation metrics number of contigs and genome size were calculated for each assembly from the assembly fasta file. BUSCOs were calculated for each assembly using BUSCO v3.0.2 (BLAST+ v2.6.0, HMMER v3.1b2 and AUGUSTUS v3.2.3), with the eukaryote_odb9 dataset and default options (-sp fly) [31].

Average and standard deviation values for the groupings presented in the tables and figures for each metric were calculated in R [32]. R was also used to construct the scatter plots for the figures.

### Hybrid assemblies

Hybrid genome assemblies were generated using the program Pilon (version 1.22) [33]. Briefly, short, highly accurate reads are mapped to a long-read assembly to correct for the higher error rate in the long reads. For each hybrid assembly, the Illumina reads were mapped using BWA to the long-read assembly. After sorting and indexing the alignments with SAMtools, pilon was run with default parameters to generate the hybrid assemblies.

The improvement of the hybrid assembly over the long-read assembly from which it was built was measured by the BUSCO scores of each (calculated as described above). BUSCO scores were preferred because they do not depend on having a reference sequence and gene finding depends on assembly accuracy. The best hybrid assembly for each isolate was deposited at DDBJ/ENA/GenBank under the accession numbers VSRS00000000 (*Giardia* beaver), VSRT00000000 (*Giardia* AWB) and VSRU00000000 (*Giardia* BGS). The versions described in this paper are versions VSRS01000000, VSRT01000000 and VSRU01000000, respectively.

### Draft annotation of hybrid genomes

Gene models were transferred from the AWB reference genome to the hybrid genomes by mapping known proteins from the *Giardia* AWB reference genome to the hybrid assembly with the program exonerate v2.2.0 [34]. Only the best match for each query protein was retained in the annotation. The draft annotations can be found in Additional files 2, 3 and 4 for *Giardia* AWB, BGS and Beaver, respectively.

### Structural variant prediction and analysis

Structural variants were predicted using the programs ngmlr and sniffles [12]. For each *Giardia* isolate, the long reads (without any correction) were mapped to the best hybrid assembly using ngmlr v0.2.7. The resulting alignments were sorted with SAMtools and the variants were called with sniffles v1.0.10.

Genes likely to be affected by the structural variants were identified by computing the overlapping regions between the genes found in the annotation step and the variant regions using BEDtools.

For each variant type, the list of putatively affected genes was examined, and genes of interest were analyzed for enrichment in the variants. For each predicted variant, 10,000 random samples of the same size as the variant were selected from the genome. For each sample the overlapping genes were found, and the genes of interest were counted. The 95th percentile was calculated from the resulting distribution of genes of interest using the nearest-rank method to find the count above which there is significant enrichment of the gene of interest (i.e. the cut-off for rejecting H_0_). The subsampling experiment was implemented in Java, the code for which is available on github at https://github.com/StephenMJPollo/SV_Subsampling.

### Genome assembly for *Giardia* beaver

The genome of *Giardia* beaver was assembled *de novo* the same as AWB and BGS hybrid assemblies described above (long-read assembly from 1D minION reads using SMARTdenovo, addition of Illumina reads to create final hybrid assembly).

## Results

### Reference quality assemblies

#### Performance of ONT long reads

The MinION sequencing runs used here produced several hundred thousand reads each with the exception of Run2, which was a second run conducted on a previously used flow cell (Table 1). In addition to producing fewer reads, re-using the flow cell also resulted in lower proportions of reads passing the quality threshold during basecalling with 64% and 81% of 1D reads passing in Run2 compared to 90–98% of 1D reads passing in Runs 1, 3 and 4 (Table 1). NanoOK [26] analysis of read error profiles showed that reads from Run2 have lower aligned base identity, higher substitutions per 100 bases, and higher indels per 100 bases compared to the other runs (Table 2).

**Table 2:** Read error profiles for *Giardia* AWB and *Giardia* BGS MinION sequencing runs

Error type/reads	AWB_0150	AWB_0157	AWB_2331	AWB_2338	BGS_2237	BGS_2244
Proportion of reads counted (%)	87.55	83.56	28.04	52.61	12.62	77.47
Overall base identity (%)	76.907	74.577	54.293	65.904	58.255	56.636
Overall base identity error rate (%)	23.093	25.423	45.707	34.096	41.745	43.364
Aligned base identity (%)	90.526	89.352	83.076	83.915	91.429	89.954
Aligned base identity error rate (%)	9.474	10.648	16.924	16.085	8.571	10.046
Identical bases per 100	80.430	78.338	71.024	71.597	80.855	78.834
Inserted bases per 100	5.291	3.881	7.811	5.087	3.473	4.478
Deleted bases per 100	5.860	8.450	6.758	9.592	8.105	7.886
Substitutions per 100	8.415	9.334	14.406	13.725	7.569	8.801
Mean insertion	1.638	1.462	1.755	1.480	1.482	1.530
Mean deletion	1.621	1.787	1.591	1.788	1.848	1.898

*Notes*: Using NanoOK [26], 1D reads were aligned to the corresponding reference genome and the error profiles of aligned reads were evaluated. NanoOK outputs read error profiles for each reference contig. To obtain overall error profiles for all reads, the values for each contig were multiplied by the proportion of total reads that aligned to that contig. The sum of these values for each error metric were scaled according to the proportion of total sequencing reads that were used for NanoOK’s analysis

NanoOK analysis of 1D read error profiles for all runs indicated a 9–17% error rate in the regions of reads that aligned to the reference genome (Table 2, aligned base identity) and a 24–46% error rate across the entirety of reads that aligned to the reference genome (Table 2, overall base identity). The analysis also showed more deleted bases than inserted bases in the reads (Table 2). Average and maximum read lengths for all runs are presented in Table 1. Notably, the maximum 1D read length generated in the sequencing runs analyzed here was 1,132,445 bases, though this read did not align to any *Giardia* reference genome nor did it have significant BLAST hits longer than ~ 45 bp in the nr database (data not shown). It is presumably a strand that got stuck but continued to generate (incorrect) sequence data.

**Fig. 1:**
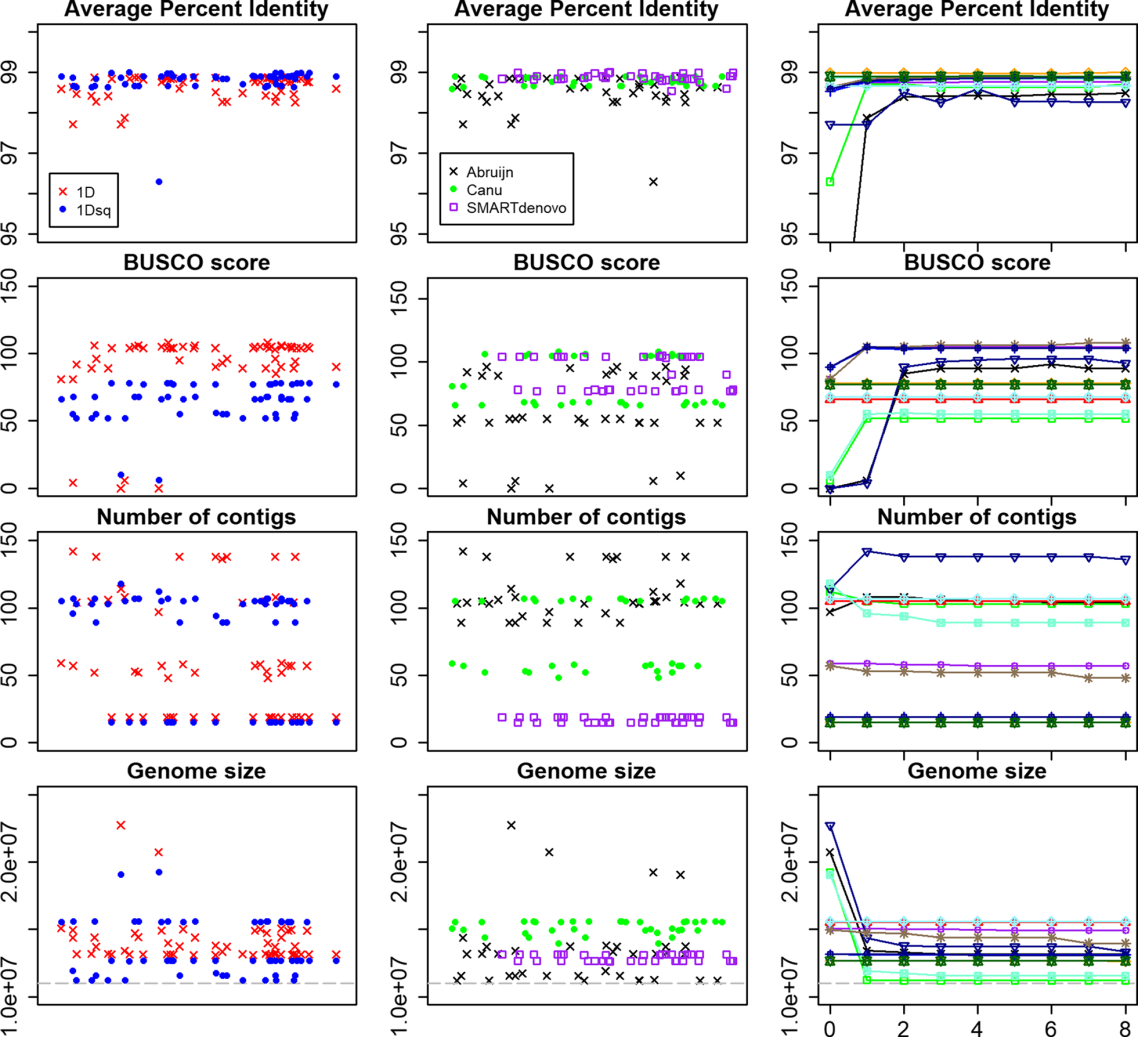
Performance metrics for all *Giardia* BGS long-read assemblies. The title above each scatterplot denotes the metric being plotted on the y-axis. The left column shows the differences between 1D (red Xs) *vs* 1Dsq (blue circles) data for each assembly protocol. Note that the data are paired. The middle column shows the assemblies separated by assembly program: abruijn (black Xs), canu (green circles) and SMARTdenovo (purple boxes). In the left and middle columns, the assemblies are randomly assigned along the x-axis for visualization purposes, hence there are no units. The right column shows polished sets of assemblies with the x-axis denoting how many times the draft assembly was polished. The dashed grey line shows the size of the *Giardia* BGS reference assembly

Results of the long-read assemblies are provided in Additional file 5: Text S2 and Fig. 1, which shows the effects of 1D *vs* 1Dsq input reads, assembly program and number of genome-polishing iterations on BGS assemblies for four of the metrics.

#### Hybrid assemblies

**Fig. 2:**
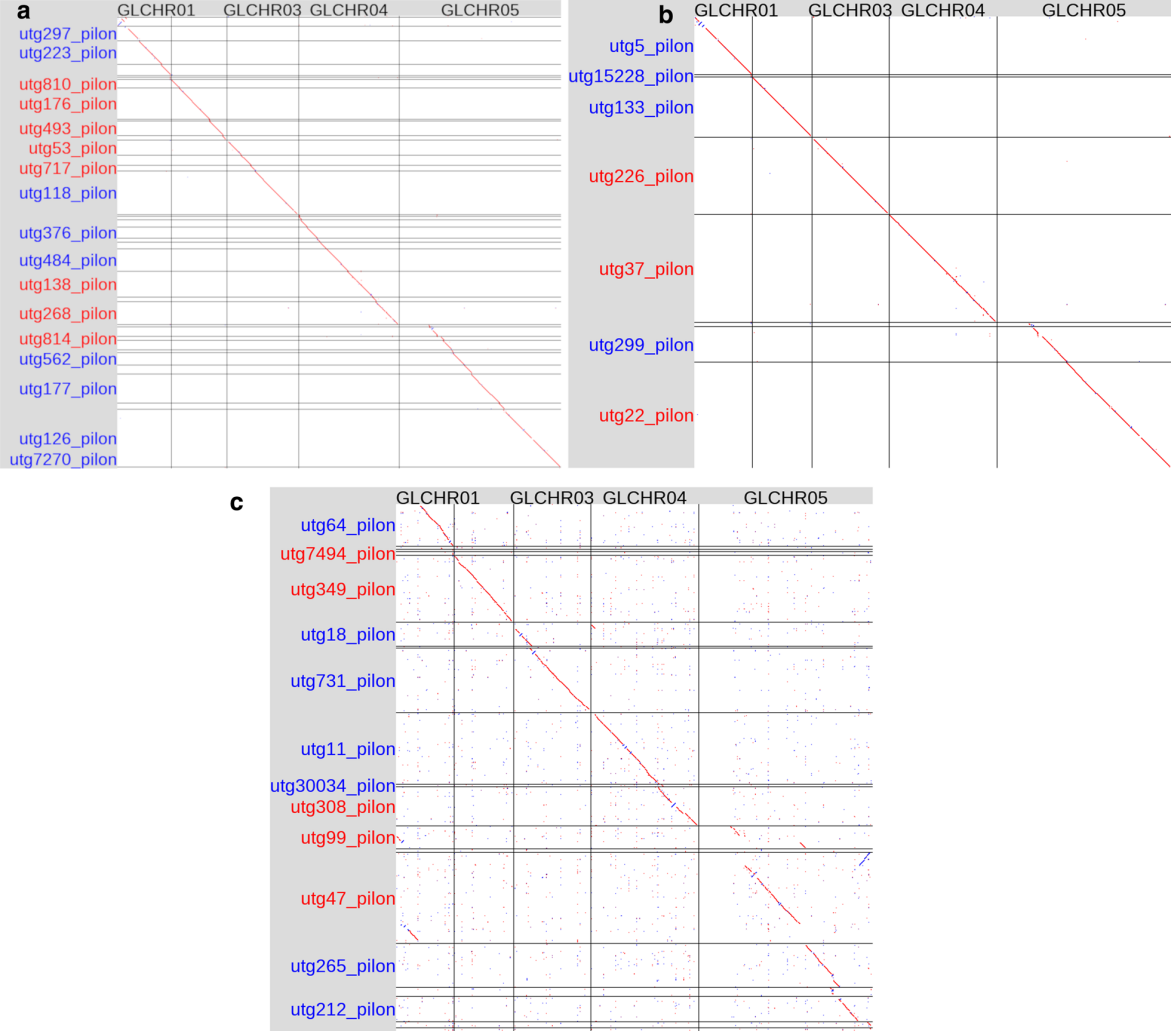
Dotplots (Oxford Grids) of pairwise whole genome alignments between the *Giardia* AWB reference genome and the *Giardia* AWB hybrid genome (**a**), the *Giardia* beaver hybrid genome (**b**) and the *Giardia* BGS hybrid genome (**c**). Each of the five *Giardia* chromosomes from the reference genome is represented as a column and each contig from the hybrid genome is represented as a row. Contig names and dots in the plot coloured red represent forward alignments while contig names and dots coloured in blue are reverse alignments

Hybrid assemblies for *Giardia* AWB were created from every AWB long-read assembly in Additional file 6: Table S1. All of the AWB hybrid assemblies with the highest complete BUSCO score (117, Additional file 6: Table S2) were constructed from a SMARTdenovo long-read assembly. For this reason and because of the performance of the long-read SMARTdenovo assemblies in general (see Additional file 5: Text S2, discussion of long-read assemblies), the *Giardia* BGS and beaver hybrid assemblies were constructed from Illumina reads and the SMARTdenovo assemblies of the 1D MinION reads. The AWB hybrid assemblies outperformed their long-read counterparts in all metrics measured (Additional file 6: Tables S1 and S2) and, for all three isolates, the hybrid assemblies had higher complete BUSCO scores than their corresponding long-read assembly. The best hybrid assembly for each isolate was selected for all further analysis on the basis of maximum complete BUSCO score (AWB_hybrid_106_0150015723312338_1dsmartx0, BGS_hybrid_gs3-20-2019_22372244_1dsmartx0, Beaver_hybrid_107218_2309_1dsmartx0). For each of these assemblies, alignment to the AWB reference genome showed that the full chromosome was recovered for chromosomes 1–4 and the majority of chromosome 5 was also recovered (Fig. 2). Transfer of gene models from the AWB reference genome to each of the hybrids resulted in 9639, 7234, and 9647 transferred genes in the AWB, BGS and beaver hybrid genomes, respectively.

### Structural variant analysis

**Fig. 3:**
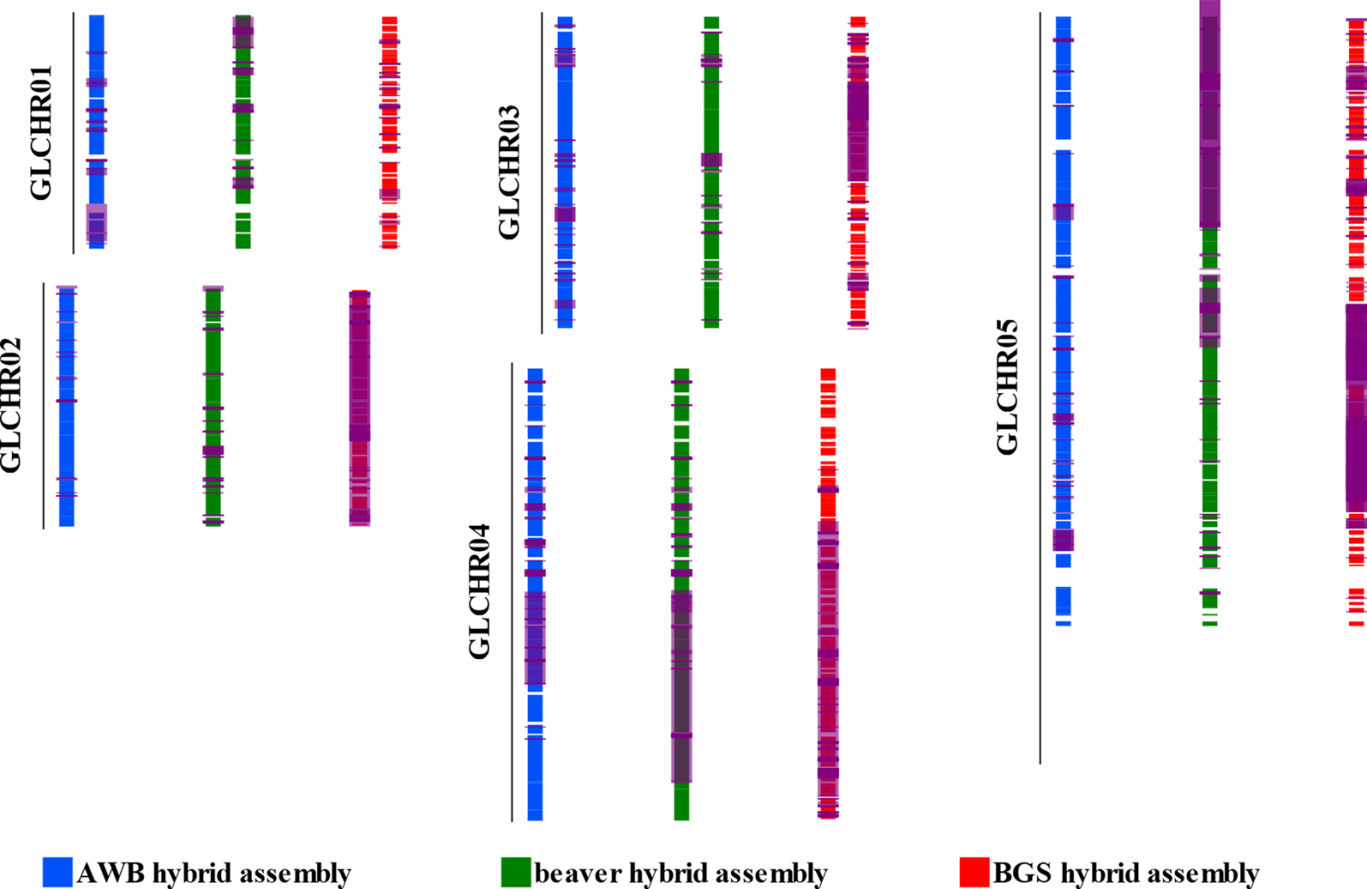
Whole genome alignments with predicted structural variants. The hybrid assembly contigs are shown as coloured boxes next to the reference *Giardia* AWB chromosome to which they align (black lines with vertical names beside each). Translucent purple boxes above the contigs show the locations and sizes of predicted structural variants in all three hybrid genomes. An interactive version of this figure with filtering capabilities can be found at: https://github.com/StephenMJPollo/Giardia_Nanopore

We predicted structural variants from the long reads and hybrid assemblies to examine the variation between the four copies of each chromosome in the *Giardia* isolates sequenced. *Giardia* AWB, BGS and beaver had 392, 1860 and 483 variants, respectively (Table 3), which affect 2072, 4151 and 3423 genes, respectively. For each isolate, the full lists of predicted structural variants and genes affected by each variant can be found in Additional file 6: Tables S3–S5. Notably among the genes affected are known virulence factors including variant-specific surface proteins (VSP), tenascins and high cysteine membrane proteins [35]. In AWB, BGS and beaver 39, 97 and 56 of the structural variants were found to have significantly more VSP than expected, respectively. Figure 3 shows alignments of the three hybrid genomes to the AWB reference genome with the predicted structural variants for each genome.

**Table 3:** Structural variants (SVs) in *Giardia* AWB, BGS and beaver

SV Property	AWB	BGS	Beaver
No. of SVs	392	1860	483
No. of duplications	45 (14,520.4)	185 (48,239.6)	69 (37,535.0)
No. of deletions	46 (15,487.1)	298 (34,454.6)	74 (46,361.1)
No. of inversions	162 (19,437.9)	746 (28,782.2)	234 (12,866.7)
No. of inverted duplications	2 (2257.0)	14 (2680.1)	0 (0.0)
No. of transversions	104 (2.3)	436 (20.8)	46 (4.0)
No. of insertions	33 (299.6)	181 (596.4)	60 (286.9)
Proportion of genome contained in SVs	0.1876	0.5662	0.3372
No. of genes in SVs	2072	4151	3423

*Note*: Numbers in parentheses are average lengths (bp) of the variants

### Genome of *Giardia* beaver

The genome of *Giardia* beaver was assembled into 8 contigs totalling 11,467,485 bp (Table 4). It has a maximum contig length of 2.759 Mb and an N50 of 1.965 Mb (Table 4). One hundred thirteen complete BUSCOs were found out of 134 detected across the three *Giardia* isolates examined here (Table 4). *Giardia* beaver has 49.56% GC content, similar to values found for *Giardia* AWB (49.0%) and other assemblage A isolates (49.25% and 49.04%; [2, 36]).

**Table 4:** Features of the three final hybrid assemblies that were submitted to GenBank

Assembly feature	*Giardia* AWB hybrid	*Giardia* BGS hybrid	*Giardia* beaver hybrid
Genome size	11,696,115	13,164,248	11,467,485
N50 (kb)	616.181	1645.00	1965.00
No. of contigs	37	19	8
Maximum contig length (Mb)	1.573	2.326	2.759
No. of complete BUSCOs	117	117	113
%GC	49.52	49.19	49.56
No. of transferred gene models	9639	7234	9647

Transfer of gene models from the AWB reference genome to the *Giardia* beaver hybrid genome resulted in 9647 transferred genes (Table 4), 3423 of which lie in predicted structural variant regions (Table 3). Roughly one third of the genome was found to be in structural variant regions, most of which are inversions, though the longest variants tend to be deletions and duplications (Table 3).

## Discussion

### Long-read assemblies and assemblers that lead to reference quality hybrid assemblies

Among the three assemblers tested, the SMARTdenovo assemblies for both *Giardia* AWB and BGS showed the lowest variability in all metrics except average indel size (Fig. 1, Additional file 7: Figures S1–S10). Moreover, the SMARTdenovo assemblies had the highest average values for average percent identity, BUSCO score and proportion of reference covered 1× (where higher values indicate better performance) (Additional file 6: Table S1) and consistently strong performance in all metrics except average indel size (Fig. 1, Additional file 7: Figures S1–S10). Despite thirteen of the top performing assemblies (8 AWB, 5 BGS) being Abruijn assemblies (Additional file 6: Table S6), plotting values for each metric showed Abruijn had the most variable performance (Additional file 6: Tables S7, S8, Additional file 7: Figures S1–S10). Canu assemblies generally performed somewhere between the SMARTdenovo and Abruijn assemblies (Additional file 6: Tables S7, S8).

Analysis of the 207 AWB and 108 BGS assemblies indicates that the optimal long-read only assembly pipeline for MinION sequenced *Giardia* is a SMARTdenovo assembly from 1D reads (either pooled or non-pooled input to reach sufficient genome coverage) followed by four or five rounds of polishing with Nanopolish (see Additional file 5: Text S2, Additional file 6: Tables S9–S15, for discussion of 1D *vs* 1Dsq input reads, pooling different sequencing runs for the same organism and number of rounds of genome polishing). However, it was the unpolished long-read assemblies that resulted in the best hybrid assemblies (1D read, SMARTdenovo assembled, no polishing with Nanopolish; Additional file 6: Table S2). Interestingly, the BGS assemblies are larger than the reference BGS assembly that was generated from 454 data [4], potentially due to the fragmented nature of the reference assembly. The AWB and BGS hybrid assemblies generated here have higher complete BUSCO scores than the available reference genomes (117 for both hybrids *vs* 114 AWB reference and 116 BGS reference) and were assembled into very large pieces (AWB hybrid N50: 616 kb; BGS hybrid N50: 1645 kb), suggesting they are of reference quality (Figs. 2, 3). Moreover, the hybrid genome for *Giardia* beaver has a similarly high complete BUSCO score and similar contig numbers and contig lengths to the AWB and BGS hybrids, indicating that reference quality assemblies can be generated *de novo* for *Giardia* with as little as one ONT MinION and one multiplexed Illumina MiSeq sequencing run. Most of the gene models from the AWB reference genome (9755) transferred to the hybrid genomes (Table 4), highlighting the completeness of the hybrid assemblies.

Each new release of a program specializing in handling long error prone reads can alter the optimal assembly pipeline for MinION data, but having the scripts to calculate the evaluation metrics used here enables rapid re-evaluations of assembler performance that could keep pace with software development. While the typical publication process, from numerous drafts of a manuscript and peer-review, cannot keep up with software development, a blog or community forum similar to an analysis on github of MinION basecalling programs [37] can and would therefore be more appropriate. These media also facilitate discussion on issues surrounding installation of programs and running them in various computing environments (e.g. some of the programs used here took up to a month to get installed and running properly). Combined with a current analysis of available long-read assemblers, such a forum would also allow researchers to determine which programs are worth the time to get working and when it may be a better use of time to go with programs that need less configuration (like Canu which worked immediately) but will still perform adequately for the intended purpose.

### Structural variants reveal different levels of intra-isolate variation

Despite having similar genome sizes, the three isolates examined here have very different total numbers of variants detected and proportions of their genomes that are within a structural variant region (Table 3, Fig. 3). When *Giardia* BGS was first sequenced, the authors noted a much higher allelic sequence heterozygosity than what was observed in AWB (0.53% in BGS *vs* 0.01% in AWB) [4]. The same trend is observed in the structural variants here with BGS being considerably more heterozygous than AWB. The differences in allelic sequence heterozygosity were attributed to AWB and BGS being in different assemblages [4]. While the values for *Giardia* beaver (an Assemblage A isolate) being more similar to AWB than BGS (Table 3) tentatively support the hypothesis that Assemblage B is more heterozygous than Assemblage A, many more genomes from each assemblage are needed to confirm it. Further, single cell sequencing could be used to examine the population structure of the isolates at a genetic level. Nonetheless, assemblage-specific variations in heterozygosity, or even isolate-specific variations in heterozygosity, will be important to consider in future comparisons between *Giardia* genomes. Previous genomic comparisons between assemblages [4] and within assemblages [38] have focused on SNPs and analyses of specific gene families. Including structural variant information provides a more complete picture of the heterozygosity and genetic diversity of each isolate by capturing differences in gene dosage as well as gene content.

### Effects of recombination in *Giardia* on structural variants

Recombination between different cells (outcrossing) within and between isolates of *Giardia* has been suggested to occur through an as-yet undiscovered mechanism [39–42]. Outcrossing recombination events would allow for changes in gene copy number if the event involved or encompassed a structural variant like a duplication or deletion. Alternatively, large inversions can prevent recombination in the inverted areas [43], preventing gene flow during recombination events in *Giardia*. These regions are therefore important to keep in mind in future studies on recombination in *Giardia* as they may confound the analyses. Several dozen structural variants from each of the isolates examined here were found to be significantly enriched for VSP, supporting the suggestion that recombination is a potential source of VSP variation [44]. Expansions and contractions of this gene family through inheritance during outcrossing events of duplicated or deleted loci that affect VSP could be an important factor in the number and distribution of these genes between the various *Giardia* assemblages and isolates. As key surface proteins involved in host immune evasion [45], these expansions and contractions of the VSP repertoire could partially explain differences in pathogenicity between isolates. Moreover, as mediators of the *Giardia* cell’s interaction with its surrounding environment, expansions and contractions of the VSP repertoire could affect host range. Alternatively, these genes could be hotspots for recombination events that generate structural variants. Then in addition to their roles as surface proteins they would also be potential factors influencing the evolution of *Giardia* genomes.

## Conclusions

The present study demonstrates that high quality genomes can be generated for *Giardia* for a few thousand dollars per genome, thus enabling future large-scale comparative genomic studies of the genus. Moreover, third-generation long reads can be further used to investigate heterozygosity and genome organization in *Giardia* despite its tetraploidy. We showed that structural variant regions affect many genes notably virulence factors including VSP, suggesting an important mechanism in the inheritance and distribution of these proteins among *Giardia* isolates. Finally, we have generated a reference genome sequence for a new isolate, *Giardia* beaver, with accompanying prediction of its structural variants.

## Supplementary information

**Additional file 1: Text S1.** Plaintext of commands used in analyses.

**Additional file 2:** Draft annotation (gff format) of AWB hybrid genome.

**Additional file 3:** Draft annotation (gff format) of BGS hybrid genome.

**Additional file 4.** Draft annotation (gff format) of beaver hybrid genome.

**Additional file 5: Text S2.** Additional results and discussion on long-read assemblies.

**Additional file 6: Table S1.** All metrics for AWB and BGS long-read assemblies. **Table S2.** All metrics for AWB hybrid assemblies. **Table S3.** Overlapping structural variants and genes in AWB. **Table S4.** Overlapping structural variants and genes in BGS. **Table S5.** Overlapping structural variants and genes in *Giardia* beaver. **Table S6.** Top performing AWB and BGS long-read assemblies. **Table S7.** Summarized assembler metrics in AWB long-read assemblies. **Table S8.** Summarized assembler metrics in BGS long-read assemblies. **Table S9.** Summarized metrics for 1D *vs* 1Dsq AWB long-read assemblies. **Table S10.** Summarized metrics for 1D *vs* 1Dsq BGS long-read assemblies. **Table S11.** All metrics for corresponding 1D and 1Dsq AWB and BGS long-read assemblies. **Table S12.** Summarized metrics for pooling input AWB long-read assemblies. **Table S13.** Summarized metrics for pooling input BGS long-read assemblies. **Table S14.** Summarized metrics for polishing AWB long-read assemblies. **Table S15.** Summarized metrics for polishing BGS long-read assemblies.

**Additional file 7: Figure S1.** 1D and 1Dsq AWB long-read assembly performance. **Figure S2.** Corresponding 1D and 1Dsq AWB long-read assembly performance. **Figure S3.** AWB long-read assembly performance by assembly program. **Figure S4.** Pooling input AWB long-read assembly performance. **Figure S5.** Performance of polishing AWB long-read assemblies. **Figure S6.** 1D and 1Dsq BGS long-read assembly performance. **Figure S7.** Corresponding 1D and 1Dsq BGS long-read assembly performance. **Figure S8.** BGS long-read assembly performance by assembly program. **Figure S9.** Pooling input BGS long-read assembly performance. **Figure S10.** Performance of polishing BGS long-read assemblies.

## Data Availability

The datasets generated and analysed during the present study are available in the SRA under the accession number PRJNA561185. The hybrid assemblies generated are available from GenBank under the accession numbers VSRS00000000 (*Giardia* beaver), VSRT00000000 (*Giardia* AWB) and VSRU00000000 (*Giardia* BGS). The versions described in this paper are versions VSRS01000000, VSRT01000000 and VSRU01000000, respectively. All other supporting materials are included in this published article and its additional files and can also be found at https://github.com/StephenMJPollo/Giardia_Nanopore. The software that runs the genome sampling can be found at https://github.com/StephenMJPollo/SV_Subsampling (Project name: SV_Subsampling; Project home page: https://github.com/StephenMJPollo/SV_Subsampling; Archived version: 10.5281/zenodo.3445450; Operating system: Linux; Programming language: Java; Other requirements: BEDtools. License: GNU GPLv3).

## Abbreviations

BUSCO: benchmarking universal single copy orthologs
ONT: Oxford Nanopore Technologies
SNPs: single nucleotide polymorphisms
SRA: sequence read archive
SVs: structural variants
VSP: variant-specific surface proteins
